# Supplementary material for: Discovery of physiological and cancer-related regulators of 3′ UTR processing with KAPAC
Source: Genome Biol. 2018 Mar 28;19:44. doi: 10.1186/s13059-018-1415-3 (PMC5875010; doi:10.1186/s13059-018-1415-3)
Supplement: Supplementary file 1 — Supplementary Figures S1–S13, Supplementary methods, Supplementary Tables S1–S5. (PDF 2.22 mb) [file 13059_2018_1415_MOESM1_ESM.pdf]

Supplementary Materials

Andreas J. Gruber[1,*], Ralf Schmidt[1,*], Souvik Ghosh[1], Georges Martin[1], Andreas R. Gruber[1], Erik van Nimwegen[1] & Mihaela Zavolan[1,§]
1 Biozentrum, University of Basel, Klingelberstrasse 50-70, CH-4056 Basel, Switzerland
∗ These authors contributed equally to this work.
§ To whom correspondence should be addressed (mihaela.zavolan@unibas.ch)

1 Supplementary Figures

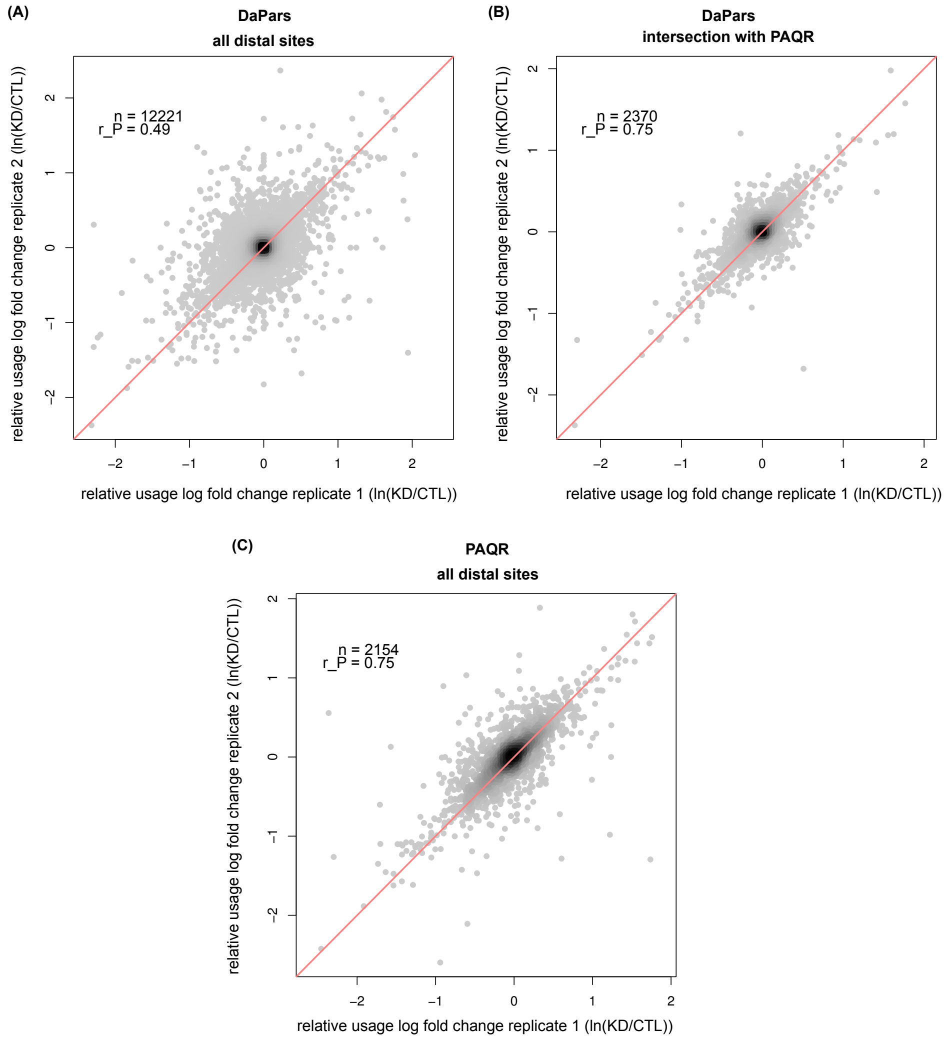

Figure S1. DaPars estimates usage of many putative PAS at the cost of accuracy. **(A)** Reproducibility of PAS usage changes in replicates of HNRNPC knock-down compared to control samples computed based on DaPars PAS usage estimates. **(B)** The reproducibility strongly increases when only sites that are also quantified by PAQR are considered (number of sites differs slightly, as sometimes, multiple DaPars sites are closely spaced and considered as one site by PAQR. **(C)** Reproducibility of PAS usage changes in replicates of HNRNPC knock-down compared to control samples computed based on PAQR PAS usage estimates.

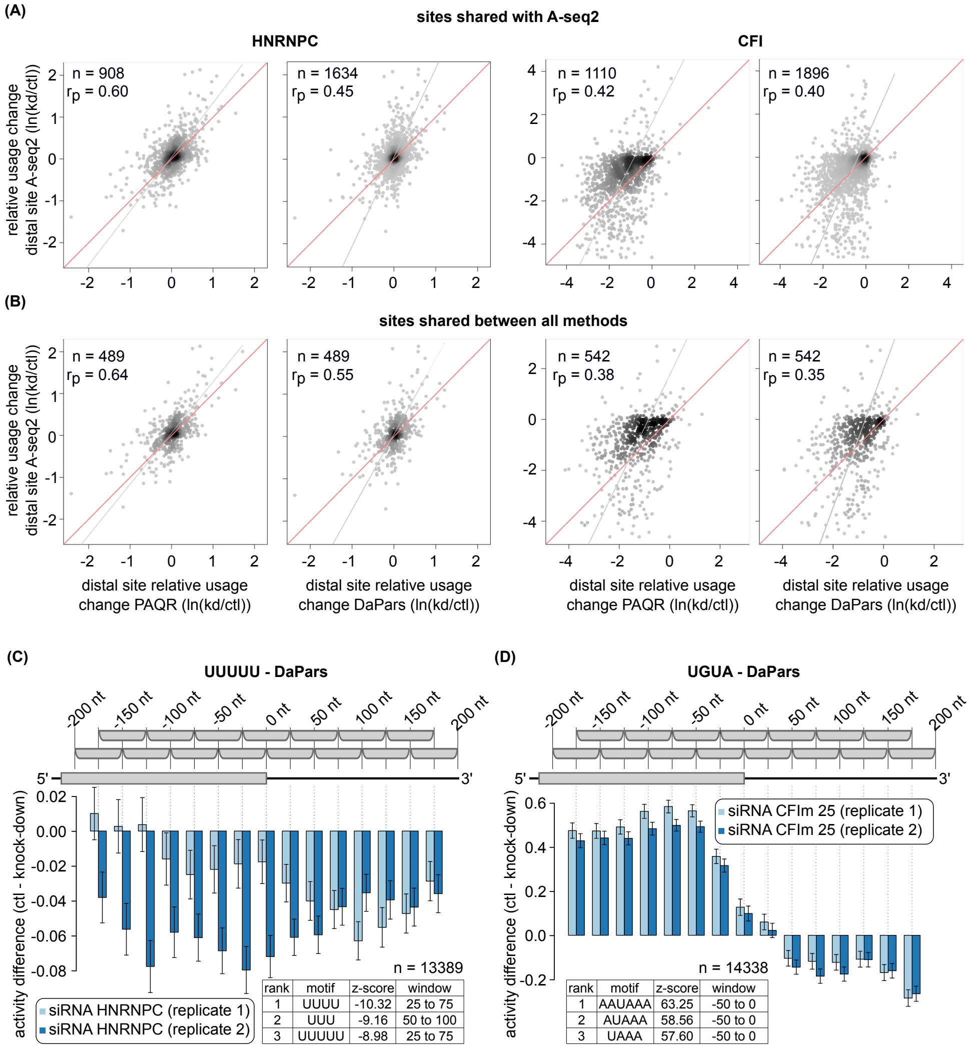

Figure S2. Accuracy of estimates of relative PAS usage by DaPars and PAQR. **(A)** Log-fold changes in the relative usage of the distal poly(A) sites in the HNRNPC knock-down experiment [1] and a CFIm 25 knock-down experiment [2] were calculated with DaPars and PAQR. The number of quantified sites that overlapped with the set of distal PAS quantified by A-seq differed between the methods. The Pearson correlation coefficients were always positive and significant but were larger for the PAQR-based quantification. **(B)** This is also the case when focusing on the PAS quantified by all three methods. **(C)** PAQR-based quantification of PAS usage yields more significant and reproducible KAPAC-inferred motif activities compared to the DaPars-based quantification. Shown is the profile of the UUUUU motif which was ranked highest based on the A-seq quantifications, and third based on DaPars quantifications. **(D)** KAPAC-inferred motif activity profile for UGUA, the binding motif of CFIm, which was ranked 13th, in the KAPAC analysis based on the DaPars PAS usage quantifications of control and CFIm 25 knock-down samples [2]. Note also that with DaPars-based quantification the motif activity remain positive also in the region downstream of PAS, which was not the case when KAPAC used A-seq-based quantification.

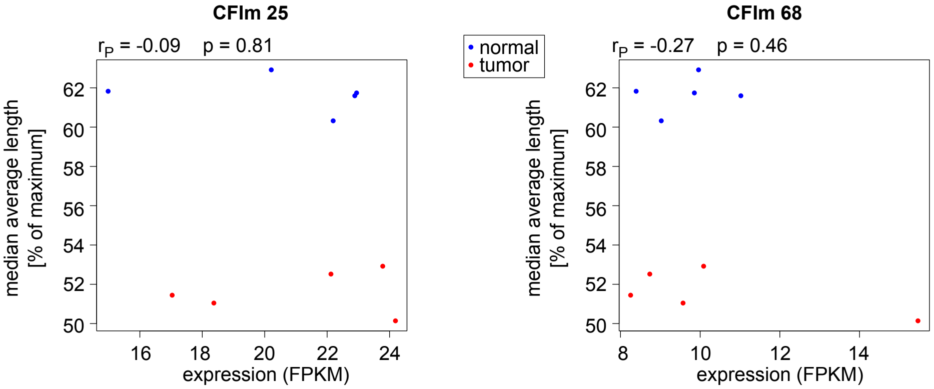

Figure S3. CFIm 25 and 68 expression estimates (in FPKM) and corresponding average exon lengths of the ten selected GBM samples.

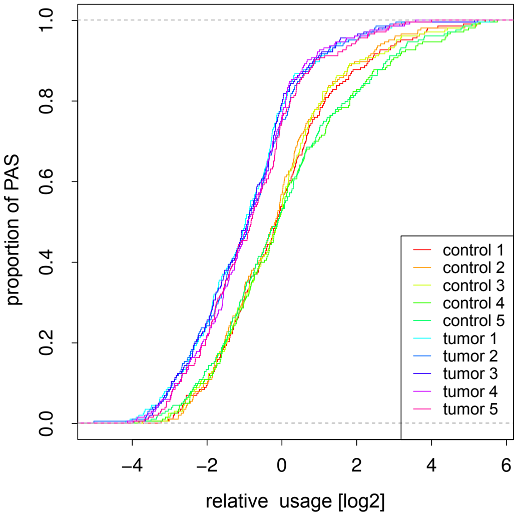

Figure S4. Analysis of PTBP1 activity in CPA in glioblastoma. Cumulative densitity functions of relative usage of the 203 PAS inferred by KAPAC to be targets of the PTBP1-binding UCUC motif in glioblastoma. The CDFs are shifted to the left in tumors, indicating decreased relative usage of the sites carrying the PTBP1-binding motif when the regulator has high expression (see Figure 5 in the main text).

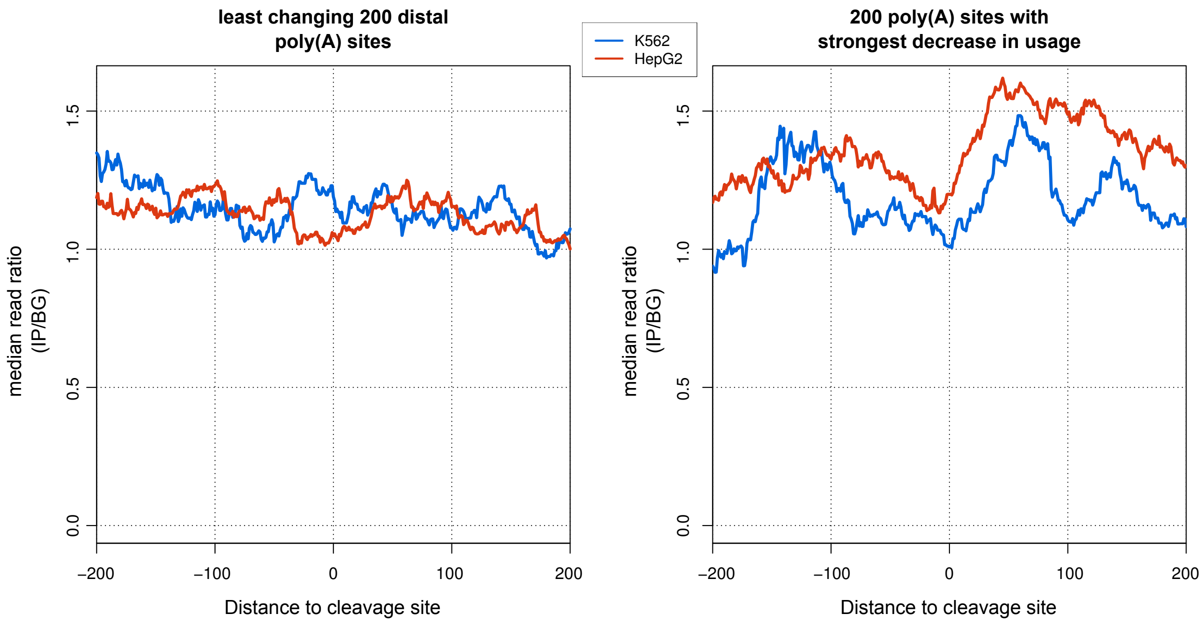

Figure S5. Position-dependent densities of PTBP1-eCLIP reads observed in two studies (in the HepG2 (thick red line) and K562 (thick blue line) cell lines). **(A)** Median per-nucleotide ratio of eCLIP read densities from the foreground (PTBP1-IP) and the background (size matched control) samples in regions around 200 distal poly(A) sites that changed least between GBM and normal tissue samples (mean change between selected random pairs of tumor-normal samples). **(B)** Same as (A) but for the 200 poly(A) sites with the strongest mean decrease in usage in GBM tumors compared to normal brain samples.

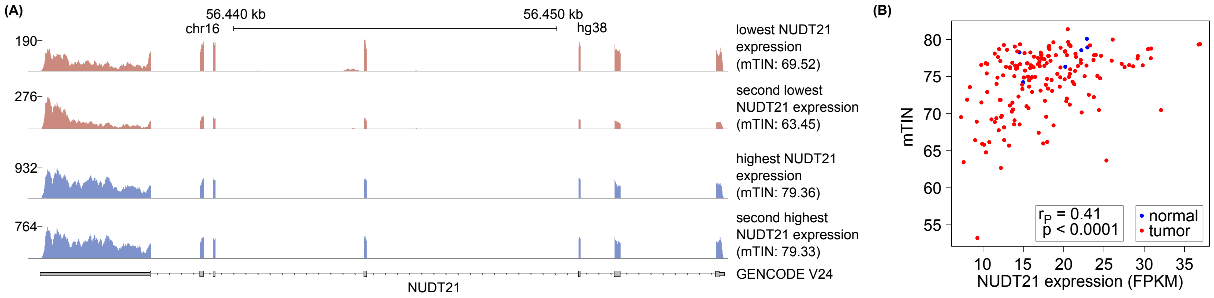

Figure S6. Partial RNA degradation can lead to apparent reduction in gene expression level. **(A)** Screen shot of the CFIm 25 (NUDT21) locus coverage in 4 different RNA-seq tumor samples from the TCGA glioblastoma cohort, shown in the IGV [3] browser. The samples were selected based on their CFIm 25 expression according to the FPKM values reported by the Genomic Data Commons (GDC) Data Portal (https://portal.gdc.cancer.gov/). The top two profiles (in red) are from samples with the lowest estimated CFIm 25 expression (FPKM), whereas the two profiles at the bottom correspond to samples withe highest estimated CFIm 25 expression. The median transcript integrity numbers (mTIN) computed over the entire transcriptome for the corresponding samples are also shown on the right side of the profiles. **(B)** Scatter plot of CFIm 25 gene expression estimates (FPKM) obtained the Genomic Data Commons (GDC) Data Portal (https://portal.gdc.cancer.gov/) and mTIN values [4], indicative of RNA degradation in the samples, shows the positive correlation between apparent expression level and RNA integrity. Normal tissue samples are in blue, tumor samples in red.

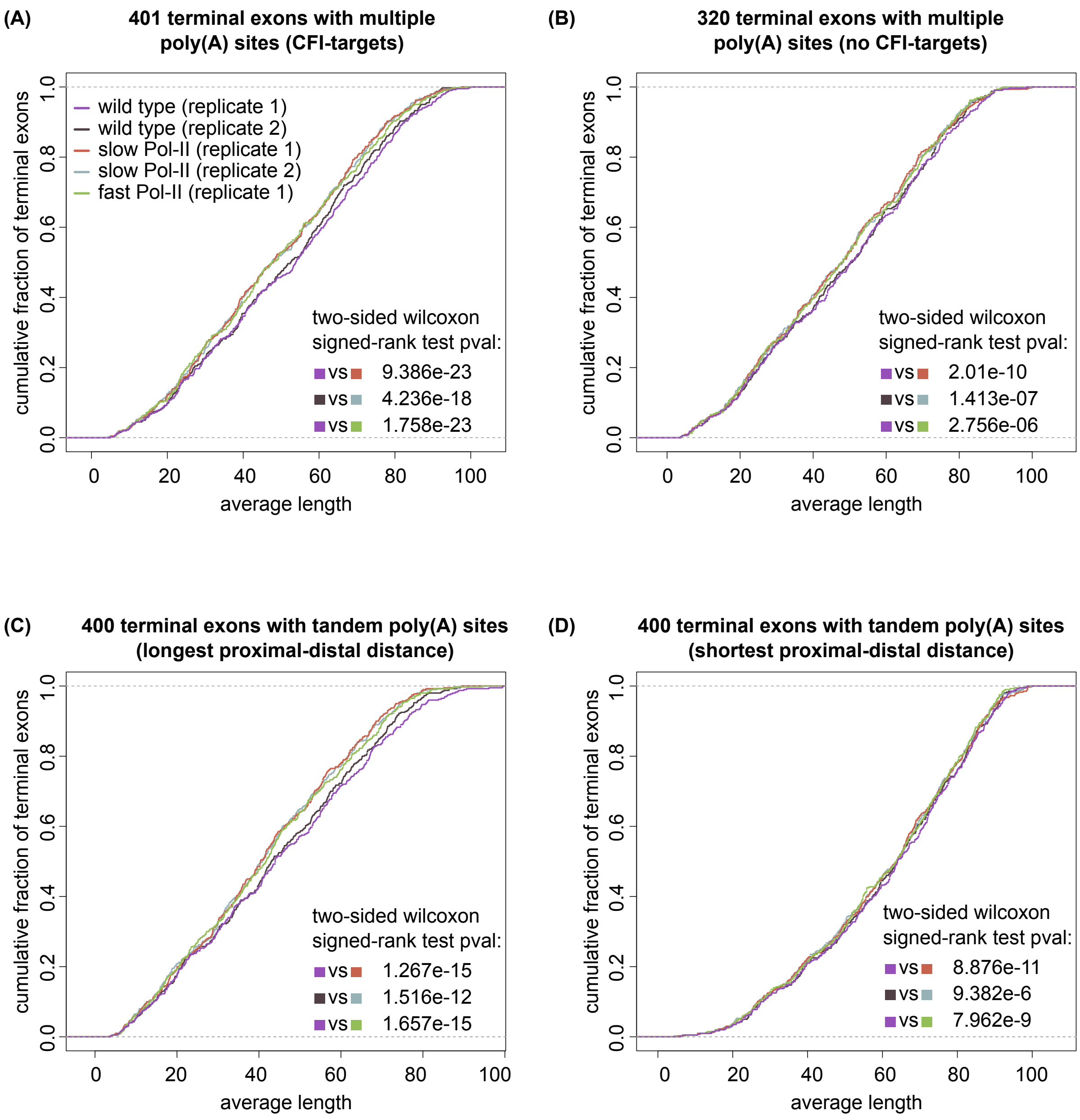

Figure S7. Cumulative distribution functions of average terminal exon length computed across the entire transcriptome from RNA-seq data sets obtained from cells expressing RNA polymerase II (RNAPII) mutants that effect the transcription elongation rate [5]. The color scheme is preserved throughout the figure. **(A)** Distributions of average length for 401 CFIm-responsive terminal exons. **(B)** Distributions of average length for a set of control exons, that did not show a large and consistent change in length upon CFIm knock-down. **(C)** Cumulative density functions of average length of 400 terminal exons with the largest distance between proximal and distal poly(A) site (among all the terminal exons that we quantified). **(D)** Complementary to (C), this panel contains the average length distributions for 400 terminal exons with the smallest distance between the proximal and the distal site.

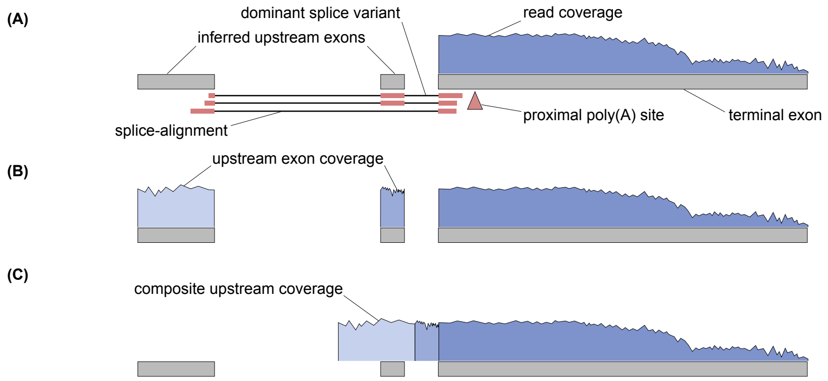

Figure S8. Schematic representation of the procedure used to compute the coverage upstream of the poly(A) site in situations when the poly(A) site is close to the start of the terminal exon. **(A)** Based on all reads that align with splicing into the terminal exon of interest, the upstream exon(s) of the major splice variant is(are) inferred. The procedure is repeated until a sufficiently long upstream exonic region is reconstructed **(B)** The read coverage profile is calculated for all inferred upstream exons. **(C)** The read coverage profile of the terminal exon is then extended upstream by the coverage profile(s) of the inferred upstream exon(s).

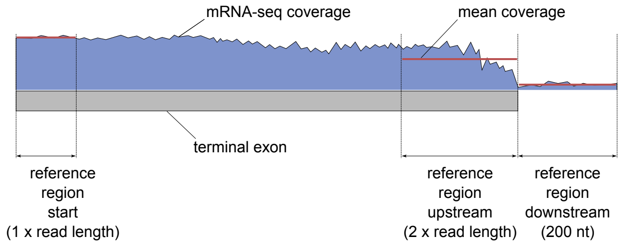

Figure S9. Schematic representation of the features used to define the most distal poly(A) site in a terminal exon.

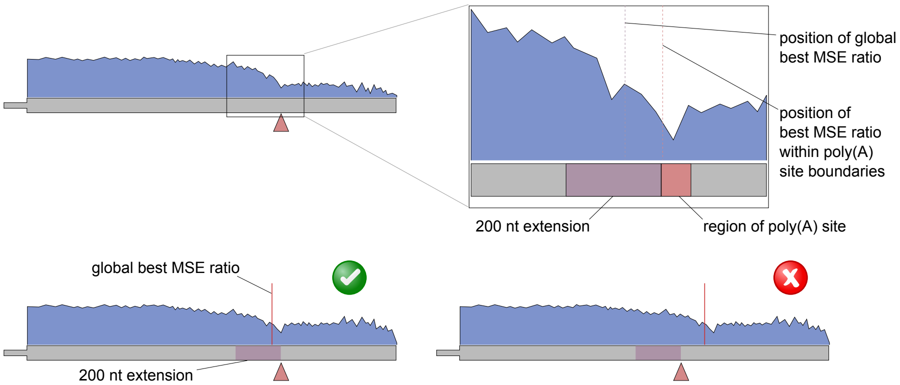

Figure S10. In a final pass of PAQR, the segmentation procedure is applied to every position within the terminal exon, identifying segments with evidence of distinct coverage. If the best segmentation point falls outside of 200 nt-long regions ending at the used poly(A) sites defined as described in the main manuscript, the exon is discarded from the analysis (bottom right panel).

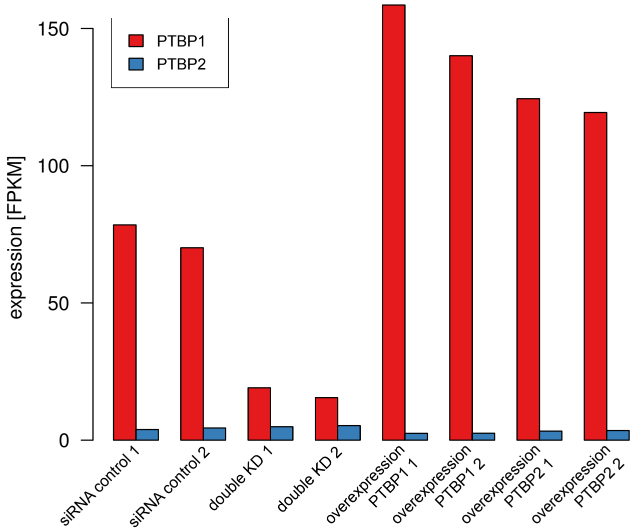

Figure S11. Expression levels of PTBP1 (red) and PTBP2 (blue) mRNAs in HEK 293 cells treated as indicated by the x-axis labels (data from [6]). These samples were used to infer PTBP1/2 activity in polyadenylation in a human cell line (see Figure 6 from main text).

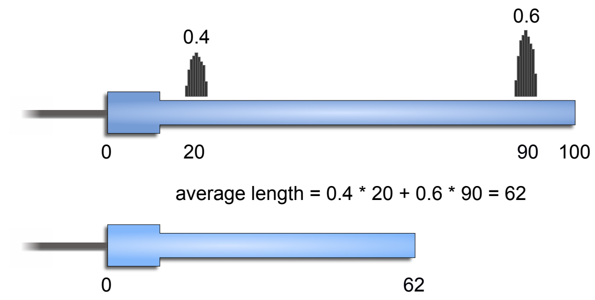

Figure S12. Schematic illustration of the calculation of average terminal exon length.

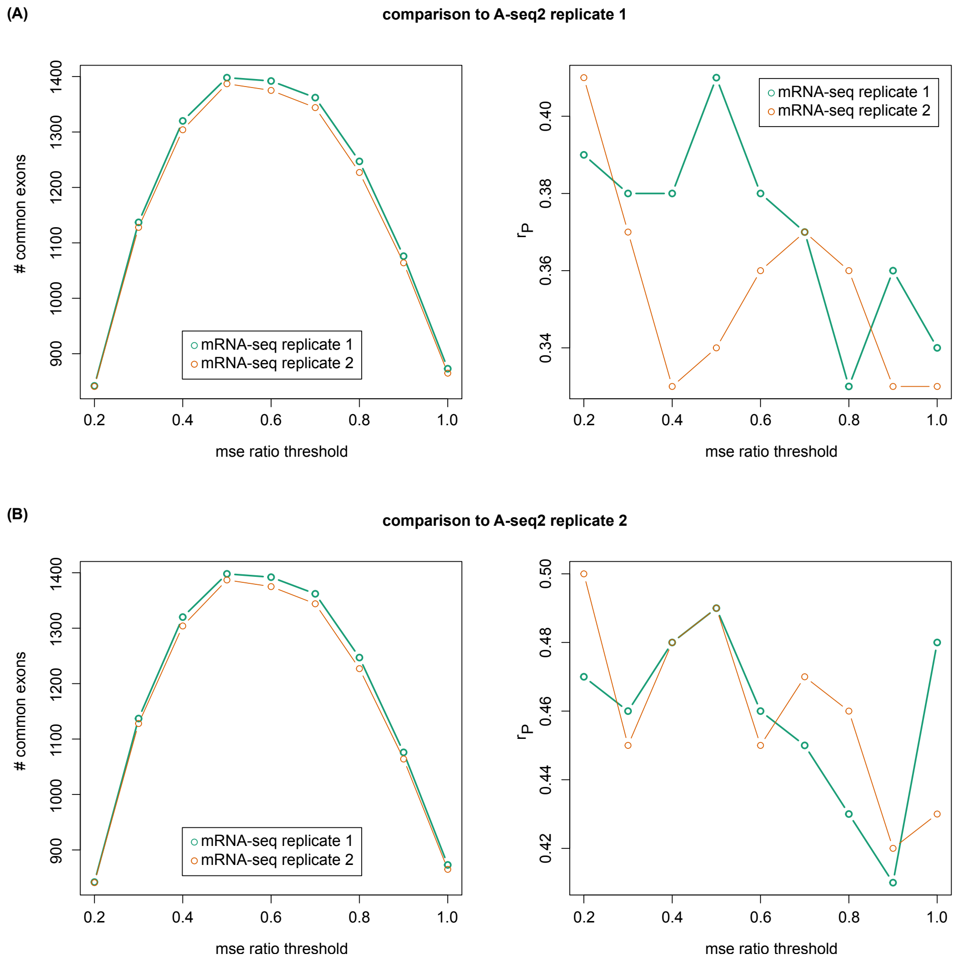

Figure S13. Comparisons of poly(A) site quantifications based on A-seq2 (SRP065825) and on RNA-seq (GSE56010) data sets. Quantifications based on RNA-seq data were done for different mean squared error (mse) ratios used to define processed poly(A) sites. **(A)** Comparisons of the A-seq2 quantifications based on samples SRX1436120 and SRX1436124 (siRNA control and si-HNRNPC replicate 1) with the RNA-seq quantifications based on GSM1502498 and GSM1502500 (replicate 1) as well as GSM1502499 and GSM1502501 (replicate 2). The left panel shows the number of terminal exons with more than one poly(A) sites for which the same set of poly(A) sites was quantified in the A-seq2 samples as well as the RNA-seq samples. For the right panel, differences in average length (between control and HNRNPC knock-down) computed for individual exons either based on A-seq2 or RNA-seq data were correlated. Shown are Pearson's correlation coefficients (r_P). **(B)** Same as shown for (A) except that the quantification of terminal exon lengths from RNA-seq data was compared with the A-seq2-based quantification (samples SRX1436128 and SRX1436130 (replicate 2)).

2 Supplementary methods

2.1 Inference of poly(A) site usage from mRNA sequencing data

The drop in coverage by RNA sequencing reads within a 3' UTR has been interpreted as evidence for an internal poly(A) site [7]. However, the read coverage per position along the 3' UTR varies quite

widely, which makes it difficult to distinguish small drops in coverage that are due to the use of proximal poly(A) sites to fluctuations that occur for unknown reasons. In contrast, the read coverage generally ends abruptly in the region downstream of the gene, which makes the distal sites easier to detect. Nevertheless, methods that exploit drops in the average coverage of 3' UTR segments to infer proximal poly(A) site usage have been developed. In particular, the DaPars method [2] has been used in multiple studies that attempted to infer regulators of polyadenylation. Attempting to quantify poly(A) site usage in two systems in which both 3' and RNA sequencing data were available, we found that DaPars quantifies a large number of putative poly(A) sites internal to 3' UTRs, of which only a very small number are deemed to have signficantly different expression changes when a specific regulator of 3' end processing is depleted by siRNA-mediated knock-down (8 of 24021 for the HNRNPC knockdown and 1265 of 26520 for the CFIm knockdown). This prompted us to develop the alternative, PAQR method, which differs from DaPars in two main aspects. First, PAQR uses an extensive database of poly(A) sites that have been determined experimentally, and does not define poly(A) sites based on the coverage profile from RNA-seq. Aside from presumably avoiding some false positive sites, this approach has the advantage that it allows us to infer position-dependent motif activities. If the location of poly(A) sites had some uncertainty, this would propagate to the location of the active motifs. Second, PAQR only aims to identify terminal exons in which there is significant usage of more than one poly(A) site. Thus, terminal exons in which internal poly(A) sites are used only to a small extent, which does not stand out of the fluctuations in coverage along the 3' UTR, are reported by PAQR as 'single poly(A) site' exons.

As shown in Supplementary Figure 2, when we compare the DaPars-based and PAQR-based quantifications of distal poly(A) site usage changes in individual exons from RNA sequencing data, with the usage changes determined by 3' end sequencing, we obtain systematically higher correlations for PAQR. This is not only due to DaPars reporting more exons, because the correlations remain higher for the set of exons that are quantified by all three methods (3' end sequencing, DaPars-based and PAQR-based quantification of RNA seq data). Consistent with the improved quantification of usage by PAQR, the absolute values as well as the significance of the motif activities that we infer with KAPAC, are higher when we use PAQR-based quantifications of poly(A) site usage than when we use DaPars quantifications (2C and D).

2.2 K-mer Activity on Polyadenylation Site Choice (KAPAC)

KAPAC, standing for K-mer Activity on Polyadenylation Site Choice, aims to identify sequence motifs (of length $k = 3 - 6$ nucleotides (nt), hence k-mers) that can explain changes in poly(A) site (PAS) use across conditions (e.g. in samples in which the expression of a potential regulator has been perturbed). It models the change in the use of alternative PAS within a transcript as a linear function of the occurrence of specific k-mers and the unknown regulatory impact (also called "activity") of these k-mers.

2.2.1 Determination of k-mer counts within defined regions relative to poly(A) sites

For a defined window relative to the poly(A) site we count the occurrences of a specific k-mer k. As we aim to identify general regulators of poly(A) site use, we discard k-mers that are found in less than 1% of all poly(A) sites. As we seek to identify factors that act in a tissue/condition-specific manner on a subset of transcripts, we calculate the number of k-mers that are found in 'excess' of what is expected to be found per chance within a sequence window (region) of interest based on the (mono)nucleotide composition of regions around poly(A) sites. Considering a region of +/-200 nt around poly(A) sites from the PolyAsite atlas (human genome hg38) [8] we have obtained the following base (B) frequencies f_B:

- Adenine: f_A=0.2973
- Cytosine: f_C=0.1935

- Guanine: f_G=0.2007
- Uracil: f_U=0.3084

Using the determined base frequencies f_B we then calculated the probability to find a k-mer k_L of length L at a specific position as follows:

$$P(k_L) = \prod_{l=1}^{L} f_{B,l} \tag{1}$$

whereat $f_{B,l}$ is the frequency of base B observed at position l. Using equation (1) we then calculated how many counts of k-mer k_L are expected to be found in a region r_W of length W given the observed base frequencies (see above):

$$N_k^e = P(k_L) * (W - L) \tag{2}$$

Finally, for a specific region (of length L) relative to a poly(A) site i we calculate the number of 'excess' counts $N_{k,i}$ by subtracting the number of expected counts of k-mer k (N_k^e) from the number of counts observed within the region $N_{k,i}^o$:

$$N_{k,i} = f^+(N_{k,i}^o - N_k^e) = \max(0, N_{k,i}^o - N_k^e) \tag{3}$$

We use the 'excess' counts determined for k-mer k within a defined region relative to poly(A) site i to explain the relative usage of the site observed within a sample s (see below).

2.2.2 Derivation of k-mer activities from genome-wide changes in poly(A) site use

We use a simple linear model that tries to explain PAS use as a function of the number of occurrences of each k-mer within a defined region in close proximity to the cleavage site and the activity of the k-mer within this region. Specifically, we define the relative use of a poly(A) site i from a terminal exon with P poly(A) sites in sample s, $U_{i,s}$ as

$$U_{i,s} = \frac{R_{i,s}}{\sum_{p=1}^{P} R_{p,s}}, \tag{4}$$

$R_{i,s}$ being the number of reads from the poly(A) site i in sample s. We relate $R_{i,s}$ to the transcription rate from the corresponding locus through the parameter α, the number $N_{i,k}$ of occurrences of k-mer k within a specific region relative to the poly(A) site i (see section 2.2.1, equation 3) and the activity $A_{k,s}$ of k-mer k within sample s:

$$R_{i,s} = \alpha * e^{N_{k,i} * A_{k,s}}. \tag{5}$$

Combining equations 4 and 5 gives:

$$U_{i,s} = \frac{e^{N_{k,i} * A_{k,s}}}{\sum_{p=1}^{P} e^{N_{k,p} * A_{k,s}}}, \tag{6}$$

or, in log-space, the relative use of poly(A) site i in sample s can be written as:

$$\begin{aligned} \log(U_{i,s}) &= \log\left(\frac{e^{N_{k,i} * A_{k,s}}}{\sum_{p=1}^{P} e^{N_{k,p} * A_{k,s}}}\right) \\ &= \log(e^{N_{k,i} * A_{k,s}}) - \log\left(\sum_{p=1}^{P} e^{N_{k,p} * A_{k,s}}\right) \\ &= N_{k,i} * A_{k,s} - \log\left(\sum_{p=1}^{P} e^{N_{k,p} * A_{k,s}}\right) \end{aligned} \tag{7}$$

We can define the mean of the log of the relative use $\langle \log(U_{t,s}) \rangle$ of a poly(A) site from terminal exon t with P_t poly(A) sites, in sample s as:

$$\begin{aligned}
\langle \log(U_{t,s}) \rangle &= \frac{\sum_{i=1}^{P_t} \log(U_{i,s})}{P_t} \\
&= \frac{\sum_{i=1}^{P_t} \left(N_{k,i} * A_{k,s} - \log\left(\sum_{p=1}^{P_t} e^{N_{k,p} * A_{k,s}} \right) \right)}{P_t} \\
&= \frac{\sum_{i=1}^{P_t} \left(N_{k,i} * A_{k,s} \right) - P_t * \log\left(\sum_{p=1}^{P_t} e^{N_{k,p} * A_{k,s}} \right)}{P_t} \\
&= \frac{\sum_{i=1}^{P_t} \left(N_{k,i} * A_{k,s} \right)}{P_t} - \log\left(\sum_{p=1}^{P_t} e^{N_{k,p} * A_{k,s}} \right) \\
&= \frac{\left(\sum_{p=1}^{P_t} N_{k,p} \right)}{P_t} * A_{k,s} - \log\left(\sum_{p=1}^{P_t} e^{N_{k,p} * A_{k,s}} \right) \\
&= \langle N_k \rangle_t * A_{k,s} - \log\left(\sum_{p=1}^{P_t} e^{N_{k,p} * A_{k,s}} \right)
\end{aligned} \tag{8}$$

where $\langle N_k \rangle_t$ is the mean count of k-mer k across the poly(A) sites of terminal exon t. We can obtain the per "terminal-exon-centered" log relative use δ_i of poly(A) site i in sample s by combining equations (7) and (8):

$$\begin{aligned}
\delta_{i,s} &= \log(U_{i,s}) - \langle \log(U_{t,s}) \rangle \\
&= N_{k,i} * A_{k,s} - \log(\sum_{p=1}^{P_t} e^{N_{k,i} * A_{k,s}}) \\
&- \langle N_k \rangle_t * A_{k,s} + \log(\sum_{p=1}^{P_t} e^{N_{k,i} * A_{k,s}}) \\
&= N_{k,i} * A_{k,s} - \langle N_k \rangle_t * A_{k,s} \\
&= (N_{k,i} - \langle N_k \rangle_t) * A_{k,s} \\
&= \bar{N}_{k,i} * A_{k,s}
\end{aligned} \tag{9}$$

where $\bar{N}_{k,i}$ are the per "terminal-exon-centered" site counts of k-mer k at poly(A) site i from terminal exon t.

As we are interested in finding k-mers that can explain changes in poly(A) site use between samples (e.g. control vs. knock-down) we finally calculate the change of the per "terminal-exon-centered" log relative use $\delta_{i,s}$ relative to the mean use across samples:

$$\begin{aligned}\bar{\delta}_{i,s} = \delta_{i,s} - \langle\delta_i\rangle &= (N_{i,k} - \langle N_k\rangle_t) * A_{k,s} - (N_{i,k} - \langle N_k\rangle_t) * \langle A_k\rangle \\ &= (N_{i,k} - \langle N_k\rangle_t) * (A_{k,s} - \langle A_k\rangle) \\ &= (N_{i,k} - \langle N_k\rangle_t) * \bar{A}_{k,s}\end{aligned} \tag{10}$$

whereas $\bar{A}_{k,s}$ is the activity of each k-mer k relative to the mean activity across samples $\langle A_k\rangle$ and $\langle\delta_i\rangle$ is the mean relative use of poly(A) site i across samples which is defined as:

$$\langle\delta_i\rangle = (N_{i,k} - \langle N_k\rangle_t) * \langle A_k\rangle \tag{11}$$

with $\langle A_k\rangle$ being the mean activity of motif k across samples.

Substituting the per "terminal-exon-centered" counts into equation (10) we find that the relative use of poly(A) site i in sample s should satisfy

$$\bar{\delta}_{i,s} = \bar{N}_{k,i} * \bar{A}_{k,s} + \epsilon \tag{12}$$

which allows us to obtain a fitted activity $\tilde{A}_{k,s}$ and a corresponding error $\tilde{\sigma}_{k,s}$ for each k-mer k in sample s, using a standard least-squares approach to solve the simple linear regression model (equation (12)).

2.2.3 Ranking of k-mers

For each pair of treatment-control samples *tcp* (or tumor versus (matched) normal tissue) we calculate for each k-mer k an activity difference z-score:

$$z_{tcp,k} = \frac{\tilde{A}_{k,control} - \tilde{A}_{k,treatment}}{\sqrt{\tilde{\sigma}^2_{k,control} + \tilde{\sigma}^2_{k,treatment}}} \tag{13}$$

We then combine the data from multiple replicates (or multiple patients) by calculating a mean activity difference z-score (Z_k) considering all treatment-control pairs:

$$Z_k = \frac{\sum_{tcp}^{TCP} z_{tcp,k}}{TCP} \tag{14}$$

where TCP is the number of treatment versus control pairs of samples (*tcp*). KAPAC ranks k-mers by their absolute mean activity difference z-scores.

2.2.4 Determination of significant mean activity difference z-scores

Given that real sequences have compositional biases that are difficult to model, we evaluate the significance of an inferred mean activity difference z-score (Z_k) for k-mer k (see equation (14)) using a randomization approach. Namely, we randomize the associations of changes in poly(A) site use with k-mer counts, by randomizing the expression values of individual poly(A) sites across the genome and then calculating the relative use of poly(A) sites according to equation (4). We fit the model, repeating the procedure (e.g. 100 times). Then, for each k-mer k, we calculate the p-value of the real mean activity difference z-score (Z_k), assuming a Gaussian distribution of the score for the k-mer, with mean and variance determined from the randomized runs. KAPAC reports the obtained p-value, the Bonferroni adjusted p-value (taking into account the total number of considered k-mers) as well as the p-value obtained by conducting a Shapiro-Wilk normality test on the mean activity difference z-scores from the randomized runs.

2.3 K-mer rankings and activity plots presented in Figures 2-6 of the main manuscript

As 3' end processing factors generally bind at defined distances with respect to the processing site, we have performed the KAPAC analysis independently for regions located at specific distances from the poly(A) sites. We have used windows of 50 nt, sliding by 25 nt at a time ('Sliding Window Approach'). A similar analysis could be implemented for windows extending to defined distances upstream or downstream of the PAS ('Extending Window Approach'). To identify the most active k-mers across all regions, we used for each k-mer the highest absolute mean activity difference z-score (Z_k, see equation (14)) across all regions, as a ranking criterion. k-mers with a Bonferroni adjusted p-value greater or equal to 0.05 were not considered (e.g. Supplementary Table 3).

2.4 Prediction of 'targets' of the CU-rich repeat motif used for the PTBP1-eCLIP data analysis

We evaluate the 'quality' of a target, containing a specific motif inferred to be active in samples of interest, by comparing the log likelihoods of the model that includes the counts $k_{i,s}$ of the motif k in the putative target region p and sample s and a model that does not. The difference in likelihoods gives us a measure of how important the motif is for predicting the observed change in the use of the respective poly(A) site.

We predict the relative use $\tilde{\delta}_{i,s}^{N}$ of a poly(A) site i in sample s from the inferred k-mer activities:

$$\tilde{\delta}_{i,s}^{N} = \bar{N}_{k,i} * \tilde{A}_{k,s} \tag{15}$$

We then calculate the $\chi_{i,s}^{2,N}$ statistic for all the poly(A) sites (P_t) in the corresponding terminal exon:

$$\chi_{i,s}^{2,N} = \sum_{t}^{P_t} (\delta_{t,s} - \tilde{\delta}_{t,s}^{N})^2 \tag{16}$$

where $\delta_{t,s}$ is the measured per "exon-centered" log relative use of poly(A) site t in sample s (see equation 9).

Next, we calculate the predicted expression using the model in which we have set the counts of k-mer k at poly(A) site i ($N_{i,k}$) to zero and then re-centered the k-mer counts for all poly(A) sites in the corresponding exon thereby obtaining a new k-mer count matrix N'. We use N' to predict the relative use $\tilde{\delta}_{i,s}^{N'}$ of a poly(A) site i in sample s using the inferred k-mer activities:

$$\tilde{\delta}_{i,s}^{N'} = \bar{N'}_{k,i} * \tilde{A}_{k,s} \tag{17}$$

Afterwards we calculate the $\chi_{i,s}^{2,N'}$ of a poly(A) site i in sample s using the new k-mer count matrix N' by summing over all poly(A) sites that are located in the exon (containing P_t expressed poly(A) sites, including i) as follows:

$$\chi_{i,s}^{2,N'} = \sum_{t}^{P_t} (\delta_{t,s} - \tilde{\delta}_{t,s}^{N'})^2 \tag{18}$$

where $\delta_{t,s}$ is the measured per "exon-centered" log relative use (as in equation 16 above).

We use the normalized log likelihood ratio as score $S_{i,k}$ for k-mer k targeting poly(A) site i:

$$S_{i,k} = \frac{\sum_s^S (\chi_{i,s}^{2,N'} - \chi_{i,s}^{2,N})}{\langle \chi^2 \rangle * P_t} \tag{19}$$

whereas P_t is the number of expressed poly(A) sites in the exon that contains poly(A) site i and the average squared-deviation per sample/poly(A) site combination $\langle \chi^2 \rangle$ is defined as:

$$\langle \chi^2 \rangle = \frac{\sum_s^S \sum_i^I (\delta_{i,s} - \tilde{\delta}_{i,s}^N)^2}{S * I} \tag{20}$$

with S being the total number of samples and I being the total number of expressed poly(A) sites.

2.5 Processing of RNA-seq data from the study of RNAPII elongation rate

Raw reads were obtained from GEO (accession number: GSE63375) and processed according to the RNA-seq pipeline for long RNAs provided by the ENCODE Data Coordinating Center (`https://github.com/ENCODE-DCC/long-rna-seq-pipeline/blob/master/dnanexus/align-star-pe/resources/usr/bin/lrna_align_star_pe.sh`) using the GENCODE version 24 human gene annotation. Based on the obtained bam files, the median TIN score (mTIN) according to Wang et al. [4] was calculated based on all transcripts with a terminal exon containing more than one poly(A) site. The obtained values were:

sample	mTIN
wild type replicate 1	78.384943
wild type replicate 2	79.868301
slow RNAPII replicate 1	77.286761
slow RNAPII replicate 2	77.233262
fast RNAPII replicate 1	79.240207
fast RNAPII repicate 2	52.603342

All samples with a mTIN score of at least 70 were further processed, i.e. replicate two of the fast RNAPII sample was not considered.

2.6 Definition of CFI targets for the analysis of RNAPII elongation rate

Only terminal exons with at least two poly(A) sites that were quantified in both studies CFIm 25/CFIm 68 knock-down in HeLa cells and RNAPII speed mutations in HEK293 cells were considered. For all samples from the CFIm study, the average length differences (knock-down/mutant versus control) were obtained and the exons were stratified as follows: exons with a consistent length change in all comparisons were used as targets whereas those with an inconsistent change in any of the comparisons were marked as non-target.

2.7 Expression estimation for PTBP1 and PTBP2

FPKM values were calculated by strictly following the GDC workflow for TCGA RNA-seq sample processing (`https://docs.gdc.cancer.gov/Data/Bioinformatics_Pipelines/Expression_mRNA_Pipeline`). After obtaining a raw read quantification of genes with HTseq-count [9], FPKM values were calculated

by using the length of a composite exon model per gene and all reads that map to protein-coding genes as library size.

2.8 Selection of distal PAS

For the comparison of DaPars [2] with A-seq, the 3' ends of the exons reported by DaPars were intersected with the poly(A) site clusters quantified by A-seq. Poly(A) site clusters that overlapped with a 3' end quantified by DaPars were considered to be expressed in both studies. Since PAQR uses annotated PAS as input, the same PAS must have been identified as distal site for the comparison of PAQR and A-seq.

2.9 Selection of subsets of poly(A) sites for the analysis of PTBP1-eCLIP read enrichment

From exons with at least two quantified poly(A) sites we selected the 200 that had the strongest mean decrease in relative usage across all GBM tumor-normal pairs. As control set, we used the 200 distal poly(A) sites whose absolute mean relative change in usage was smallest.

3 Supplementary Tables

Table S1. Overview on KAPAC results for the 3' end sequencing data of the indicated study. Shown are the 20 most significant k-mers, or all if there are less than 20 significant ones (Bonferroni corrected p-value < 0.05) with their mean activity difference z-score (between normal and tumor samples), their adjusted p-value and the window for which the activity difference z-score was inferred.

study	rank	k-mer	z-score difference	adjusted p-value	window
**PCBP1** [10]	nr 1	CCC	14.3619386027758	3.92210e-29	u75to25.d0to0
	nr 2	CCCU	12.3234804203847	1.35376e-22	u75to25.d0to0
	nr 3	UCCC	12.2883102355394	4.62113e-23	u75to25.d0to0
	nr 4	CCU	11.2584739909402	8.03581e-20	u100to50.d0to0
	nr 5	UCC	10.3078840034045	9.40719e-16	u100to50.d0to0
	nr 6	GCCC	9.26200680989259	7.40421e-10	u75to25.d0to0
	nr 7	UCCCU	8.95753524332782	6.62195e-12	u75to25.d0to0
	nr 8	CCCC	8.60555616896579	5.53819e-06	u75to25.d0to0
	nr 9	CCCUU	8.44098566788859	2.74658e-07	u100to50.d0to0
	nr 10	CCCA	7.93988407765	2.47397e-05	u125to75.d0to0
	nr 11	CCUU	7.47584995939994	3.68167e-07	u100to50.d0to0
	nr 12	UCCCC	7.37898773800494	1.23901e-03	u75to25.d0to0
	nr 13	GCC	7.15590255944904	6.66673e-04	u100to50.d0to0
	nr 14	CUCC	7.06136031907776	1.13564e-03	u100to50.d0to0
	nr 15	CUUCC	7.04848749984694	5.63203e-04	u100to50.d0to0
	nr 16	CUCCC	6.97920980000055	1.25769e-03	u100to50.d0to0
	nr 17	UGCCC	6.97713370646291	2.43523e-06	u75to25.d0to0
	nr 18	UUCCC	6.78133271365627	2.14076e-04	u75to25.d0to0
	nr 19	CCCCU	6.66612158690429	2.24487e-03	u75to25.d0to0
	nr 20	UCCCUU	6.65186476207128	1.61041e-05	u75to25.d0to0
**HNRNPC** [8]	nr 1	UUUUU	-17.1226127126567	3.13308e-43	u25to0.d0to25
	nr 2	UUU	-16.5958380846563	7.05715e-41	u25to0.d0to25
	nr 3	UUUU	-16.3028956832816	4.92083e-38	u25to0.d0to25
	nr 4	UUUUUU	-13.0554616188556	4.11552e-24	u25to0.d0to25
	nr 5	UUUG	-11.7071099513396	6.27981e-15	u50to0.d0to0
	nr 6	UUUUG	-11.1561957571474	3.77578e-14	u50to0.d0to0
	nr 7	CAG	10.627744491379	1.38687e-15	u50to0.d0to0
	nr 8	UUUUUG	-10.2783217400175	1.23036e-13	u50to0.d0to0
	nr 9	AUUUUU	-10.1232256769064	4.77752e-18	u25to0.d0to25
	nr 10	CUC	10.0776142444971	3.43206e-11	u75to25.d0to0
	nr 11	UUG	-9.97742310106386	1.53611e-10	u75to25.d0to0
	nr 12	AUUU	-9.66082399472801	1.10721e-10	u50to0.d0to0
	nr 13	GUUU	-9.5733404808427	1.22066e-11	u25to0.d0to25
	nr 14	UAUU	-9.51544130944631	4.81927e-15	u50to0.d0to0
	nr 15	UUGU	-9.43882410226739	1.50859e-12	u50to0.d0to0
	nr 16	UUUUUA	-9.37333750745204	1.73277e-09	u50to0.d0to0
	nr 17	UUUGU	-9.2243373238644	3.35869e-10	u50to0.d0to0
	nr 18	AUUUU	-9.20476745475456	5.42214e-08	u50to0.d0to0

Continued on next page

Table S1 – continued from previous page

study	rank	k-mer	z-score difference	adjusted p-value	window
	nr 19	UGU	-9.11478153686116	4.99393e-09	u75to25.d0to0
	nr 20	CUUUUU	-9.10287686716165	3.52874e-13	u25to0.d0to25
**CFIm**	nr 1	AAUAAA	10.9656674102611	4.64404e-18	u50to0.d0to0
	nr 2	UGUA	10.5588960353076	2.00776e-15	u75to25.d0to0
	nr 3	AAUAA	10.054856313557	1.39373e-14	u50to0.d0to0
	nr 4	UGU	10.0267388803685	1.84259e-14	u150to100.d0to0
	nr 5	AAUA	9.70222909873153	3.59413e-13	u50to0.d0to0
	nr 6	UAA	9.44033417374034	2.03604e-17	u50to0.d0to0
	nr 7	UAU	9.05991932725031	5.35025e-14	u100to50.d0to0
	nr 8	AAU	8.91980457038989	1.17062e-11	u50to0.d0to0
	nr 9	UAAA	8.90689935325978	2.04303e-12	u50to0.d0to0
	nr 10	UUGUA	8.86183077056082	3.07792e-14	u75to25.d0to0
	nr 11	AUAAA	8.56029151784637	9.77397e-11	u50to0.d0to0
	nr 12	UUGU	8.36396835293178	3.03781e-11	u150to100.d0to0
	nr 13	GUA	8.26380253803631	4.16344e-09	u75to25.d0to0
	nr 14	UUUA	8.23518199723387	2.24869e-08	u100to50.d0to0
	nr 15	GCC	-7.81567064349458	2.43275e-09	u100to50.d0to0
	nr 16	UUA	7.75266447353091	2.67136e-09	u100to50.d0to0
	nr 17	AAAU	7.72642936923094	7.20373e-09	u50to0.d0to0
	nr 18	UGUAA	7.64644569977783	2.41648e-08	u75to25.d0to0
	nr 19	AUAA	7.57449370048538	1.95289e-08	u50to0.d0to0
	nr 20	UUU	7.56573439493848	4.93710e-11	u150to100.d0to0

Table S2. Overview on KAPAC results for the standard RNA-seq data of the indicated study. Shown are the 20 most significant k-mers, or all if there are less than 20 significant ones (Bonferroni corrected p-value < 0.05) with their mean activity difference z-score (between normal and tumor samples), their adjusted p-value and the window for which the activity difference z-score was inferred.

study	rank	k-mer	z-score difference	adjusted p-value	window
**HNRNPC** [1]	nr 1	UUUUU	-21.66427672982	2.47991e-23	u0to0.d0to50
	nr 2	UUUU	-21.3480701671228	3.68403e-29	u0to0.d0to50
	nr 3	UUUUUU	-19.5362176459321	1.88716e-18	u0to0.d0to50
	nr 4	UUU	-17.672200613449	2.53044e-16	u0to0.d0to50
	nr 5	AUUUUU	-12.4335263034769	9.15171e-07	u25to0.d0to25
	nr 6	CUUUUU	-12.0173198238539	5.40989e-06	u0to0.d0to50
	nr 7	AUUUU	-11.9391554672639	1.96168e-08	u25to0.d0to25
	nr 8	UUUUUG	-11.1279217133331	8.46027e-06	u0to0.d25to75
	nr 9	ACUGCA	-10.4544299166344	2.85868e-05	u0to0.d75to125
	nr 10	UUUUUA	-10.3723578596203	8.66192e-06	u25to0.d0to25
	nr 11	GCCUCC	-9.96981626596616	2.17326e-05	u0to0.d100to150
	nr 12	GCCUC	-9.87551667175939	1.62913e-04	u0to0.d100to150

Continued on next page

Table S2 – continued from previous page

study	rank	k-mer	z-score difference	adjusted p-value	window
	nr 13	UUUUG	-9.65914100131002	6.08779e-04	u0to0.d0to50
	nr 14	CUUUU	-9.60735872053696	1.47678e-03	u0to0.d0to50
	nr 15	GCAACC	-9.54646549107147	3.03805e-05	u0to0.d75to125
	nr 16	UUUUUC	-9.44597128693347	2.65561e-03	u0to0.d25to75
	nr 17	GUUUUU	-9.43523677200803	9.70228e-04	u75to25.d0to0
	nr 18	CACUGC	-9.40557479481014	2.03784e-03	u0to0.d75to125
	nr 19	GCCAUU	-9.25103729882374	6.03798e-04	u0to0.d100to150
	nr 20	GCUCAC	-8.95404320524543	2.68805e-03	u0to0.d75to125
**CFIm 25** [2]	nr 1	UGU	24.3829655172491	9.18903e-28	u125to75.d0to0
	nr 2	UGUA	23.6103952635293	4.48714e-29	u100to50.d0to0
	nr 3	AUU	22.5276877058983	3.92576e-45	u150to100.d0to0
	nr 4	AAUAAA	22.1747258931642	1.70306e-29	u50to0.d0to0
	nr 5	UAU	21.6483480370493	1.63728e-26	u100to50.d0to0
	nr 6	UUGU	21.4616586590102	6.84804e-40	u150to100.d0to0
	nr 7	UUA	19.9991395007045	3.66046e-30	u150to100.d0to0
	nr 8	AAUAA	19.9668688810485	4.81553e-21	u50to0.d0to0
	nr 9	UAUU	19.9580615697656	9.55971e-30	u150to100.d0to0
	nr 10	AUUU	19.0887468345882	4.89942e-23	u150to100.d0to0
	nr 11	UUGUA	18.3972829494701	9.38891e-20	u100to50.d0to0
	nr 12	UGUAU	18.314537459356	6.23009e-19	u125to75.d0to0
	nr 13	GUA	18.2355799427803	3.93916e-19	u100to50.d0to0
	nr 14	UAA	18.1279696821075	1.78465e-15	u200to150.d0to0
	nr 15	UUUA	18.117483027468	8.87175e-23	u175to125.d0to0
	nr 16	GGA	-18.0412579969026	1.60360e-22	u150to100.d0to0
	nr 17	CAG	-17.8522835736229	4.38540e-18	u150to100.d0to0
	nr 18	AUUGU	17.7561679662	3.11635e-18	u125to75.d0to0
	nr 19	AUAAA	17.4863596662839	7.61925e-16	u50to0.d0to0
	nr 20	UUAU	17.42803060558	1.78107e-20	u150to100.d0to0
**PTBP1/2** [6]	nr 1	UCU	9.88770416432942	5.36039e-06	u25to0.d0to25
	nr 2	CUCU	8.15341786865457	1.98386e-03	u25to0.d0to25
	nr 3	CUCUCU	8.03270724839405	1.01339e-03	u0to0.d25to75
	nr 4	UUCU	7.85922178238784	3.30486e-03	u25to0.d0to25
	nr 5	UUCUC	7.51573593114661	1.49623e-02	u50to0.d0to0
	nr 6	UUGUGU	7.5029478372414	2.65028e-02	u0to0.d25to75
	nr 7	UCUUC	7.47146651882119	1.26587e-02	u50to0.d0to0
	nr 8	GACUA	7.37672158832967	1.72687e-02	u0to0.d75to125
	nr 9	UCUCCU	7.26369202083234	3.75263e-02	u50to0.d0to0
	nr 10	CUCUUC	7.16401311199207	4.13901e-02	u50to0.d0to0
	nr 11	CUC	7.04876822494446	3.88378e-02	u50to0.d0to0
	nr 12	UGGUGA	7.04005435369631	4.72596e-02	u0to0.d100to150
	nr 13	AGGACU	6.98761283234312	4.12172e-02	u0to0.d75to125
	nr 14	UUUAUA	-6.92047578472529	4.13303e-02	u25to0.d0to25

Table S3. Overview on KAPAC results for the different cancer types if they are considered in the main manuscript. Shown are the 20 most significant k-mers, or all if there are less than 20 significant ones (Bonferroni corrected p-value < 0.05) with their mean activity difference z-score (between normal and tumor samples), their adjusted p-value and the window for which the activity difference z-score was inferred.

**cancer cohort**	**rank**	**k-mer**	**z-score difference**	**adjusted p-value**	**window**
**COAD**	nr 1	UUUUU	-4.96937708701905	3.74967e-02	u50to0.d0to0
	nr 2	UUUGU	-4.21481585142658	1.73656e-02	u0to0.d150to200
**LUAD**	nr 1	AGCUUG	4.40759145275076	7.68629e-03	u75to25.d0to0
	nr 2	CCUUCC	-4.23280169144492	8.94116e-03	u0to0.d0to50
	nr 3	UAUU	4.22326523375785	5.05276e-04	u125to75.d0to0
	nr 4	GAAG	-4.04537916018301	4.83960e-02	u200to150.d0to0
	nr 5	UAU	4.02434402961917	4.09451e-03	u125to75.d0to0
	nr 6	GUAUGA	3.88754196033867	2.36437e-02	u50to0.d0to0
	nr 7	CCUUC	-3.87652295059531	3.79403e-02	u0to0.d0to50
	nr 8	GUAU	3.84571509896243	1.80215e-02	u125to75.d0to0
	nr 9	CCCAG	-3.6454244635503	3.29534e-02	u150to100.d0to0
	nr 10	CCCCUU	3.473394114303	4.78457e-02	u0to0.d50to100
	nr 11	UGUAUU	3.40476817035171	4.85372e-03	u125to75.d0to0
**PRAD**	nr 1	AUU	-4.01827015983043	2.46805e-03	u0to0.d25to75
	nr 2	AUUU	-3.99168739737411	1.26721e-02	u0to0.d25to75
	nr 3	UAUUU	-3.88652328998694	1.32727e-03	u0to0.d25to75
	nr 4	UAUU	-3.53765426692504	4.47438e-02	u0to0.d25to75
**GBM**[1]	nr 1	UCUCUC	9.90740192572668	1.68536e-06	u0to0.d25to75
	nr 2	CUCUCU	9.22208774710919	3.06294e-05	u0to0.d25to75
	nr 3	CUCUC	8.74301074486753	7.44746e-06	u0to0.d25to75
	nr 4	UCUC	8.66858168373634	4.66253e-06	u0to0.d25to75
	nr 5	UCUCU	8.35363357910616	8.08023e-04	u0to0.d25to75
	nr 6	CUC	8.07444814776459	8.29237e-04	u0to0.d25to75
	nr 7	UUAU	-7.56922933209396	4.70394e-03	u0to0.d150to200
	nr 8	CUCU	7.48320885412783	1.79984e-03	u0to0.d25to75
	nr 9	AAUAU	-7.41968885048703	3.63770e-03	u0to0.d75to125
	nr 10	AUUU	-7.39880077048514	3.89400e-03	u0to0.d50to100
	nr 11	UGUGUG	7.12420875874659	2.80547e-02	u0to0.d50to100
	nr 12	AAUCCC	6.91920737044926	1.99104e-02	u0to0.d125to175
	nr 13	AUU	-6.86277219404571	2.72602e-02	u0to0.d50to100
	nr 14	AAAUAU	-6.81585214892369	1.98374e-02	u0to0.d75to125
	nr 15	CAGGC	6.7400778189719	1.44156e-02	u0to0.d150to200
	nr 16	GGC	6.6874316792108	3.34506e-02	u0to0.d125to175
	nr 17	CAUCUU	-6.65269394378098	3.90724e-03	u175to125.d0to0
	nr 18	CUGAC	-6.64458751628684	3.93609e-04	u175to125.d0to0
	nr 19	UUA	-6.63359494853029	1.86693e-02	u0to0.d125to175
	nr 20	CCCAGC	6.29440474138774	2.49740e-02	u0to0.d125to175

[1] The given results were inferred based on randomly assigned pairs of normal and tumor samples, not on matching pairs of samples from individual patients.

Table S4. Overview on the number of considered normal-tumor comparisons and on the number of quantified terminal exons with at least two poly(A) sites per cancer cohort.

cancer cohort	number of normal-tumor comparisons	number of overall quantified exons
**BLCA**	15	2046
**BRCA**	93	2863
**COAD**	32	2470
**ESCA**	8	2338
**HNSC**	37	2779
**KICH**	19	2370
**KIRC**	63	2587
**KIRP**	24	2453
**LIHC**	13	1941
**LUAD**	58	2619
**LUSC**	46	2879
**PRAD**	43	2450
**READ**	6	1784
**STAD**	22	3208
**THCA**	37	2219
**UTEC**	9	1916

Table S5. Overall number of processed TCGA samples per cancer type (obtained from https://portal.gdc.cancer.gov/).

cancer cohort	number of samples
**BLCA**	40
**BRCA**	229
**CESC**	6
**CHOL**	18
**COAD**	87
**ESCA**	16
**GBM**	169
**HNSC**	86
**KICH**	46
**KIRC**	144
**KIRP**	62
**LIHC**	100
**LUAD**	125
**LUSC**	98
**PAAD**	8
**PCPG**	6
**PRAD**	106
**READ**	18

Continued on next page

Table S5 – continued from previous page	
**cancer cohort**	**number of samples**
**SARC**	4
**STAD**	54
**THYM**	4
**THCA**	116
**UCEC**	46

References

1. Liu, N. *et al.* N(6)-methyladenosine-dependent RNA structural switches regulate RNA-protein interactions. *Nature* **518**, 560–564 (2015).

2. Masamha, C. P. *et al.* CFIm25 links alternative polyadenylation to glioblastoma tumour suppression. *Nature* **510**, 412–416 (2014).

3. Robinson, J. T. *et al.* Integrative genomics viewer. *Nat. Biotechnol.* **29**, 24–26 (2011).

4. Wang, L. *et al.* Measure transcript integrity using RNA-seq data. *BMC Bioinformatics* **17**, 58 (2016). URL `http://dx.doi.org/10.1186/s12859-016-0922-z`.

5. Fong, N. *et al.* Pre-mRNA splicing is facilitated by an optimal RNA polymerase II elongation rate. *Genes Dev.* **28**, 2663–2676 (2014).

6. Gueroussov, S. *et al.* An alternative splicing event amplifies evolutionary differences between vertebrates. *Science* **349**, 868–873 (2015). URL `http://dx.doi.org/10.1126/science.aaa8381`.

7. Wang, W., Wei, Z. & Li, H. A change-point model for identifying 3'UTR switching by next-generation RNA sequencing. *Bioinformatics* **30**, 2162–2170 (2014). URL `http://dx.doi.org/10.1093/bioinformatics/btu189`.

8. Gruber, A. J. *et al.* A comprehensive analysis of 3' end sequencing data sets reveals novel polyadenylation signals and the repressive role of heterogeneous ribonucleoprotein C on cleavage and polyadenylation. *Genome Res.* **26**, 1145–1159 (2016).

9. Anders, S., Pyl, P. T. & Huber, W. HTSeqa python framework to work with high-throughput sequencing data. *Bioinformatics* **31**, 166–169 (2015). URL `https://academic.oup.com/bioinformatics/article-abstract/31/2/166/2366196`.

10. Ji, X., Wan, J., Vishnu, M., Xing, Y. & Liebhaber, S. A. CP Poly(C) binding proteins act as global regulators of alternative polyadenylation. *Mol. Cell. Biol.* **33**, 2560–2573 (2013). URL `http://dx.doi.org/10.1128/MCB.01380-12`.
